# Surgery

**Published:** 1838-04

**Authors:** 


### SURGERY.

### *On the Treatment of Aneurism by Ice.* By M. Reynaud, Surgeon-in-chief of the Naval Hospital at Toulon.

A seaman, aged thirty-four, became affected in the year 1833 with pains in the right groin and thigh, which were followed by the appearance of a hard, circumscribed tumour in the upper part of the thigh, which soon increased in size. When brought to Toulon the following was the report of this case. The right lower extremity is much enlarged, the circumference of the upper part of the thigh being thirty inches, whilst on the opposite side it is only fifteen. The base of the tumour extends from the anterior superior spine of the ileum to the linea alba. Its apex, wide and flattened, reaches to an inch and a half below the crural arch. In the centre there is a fluctuating point of a bluish tinge, where the skin appears so thin as to threaten an approaching rupture. The tumour is divided into two parts by the fallopian ligament, which forms a groove in the middle. Pressure does not sensibly diminish its volume. Pulsations are with difficulty perceived in the tumour; they are isochronous with the action of the heart. No pulsations are felt in the course of the femoral, the popliteal, and anterior tibial arteries. The whole limb is œdematous. The leg, semiflexed on the thigh, is incapable of motion.

From this examination it appeared evident that the common iliac was diseased as well as the external iliac and the femoral. It became therefore a question whether the operation of tying the aorta should be had recourse to; but on considering the unfortunate result of all those cases in which that operation has been hitherto employed, it was resolved to treat the case by the application of ice. The patient was accordingly put on very low diet, and a bladder containing pounded ice was constantly applied to the tumour. In four days (20th February, 1834,) the circumference of the limb diminished four inches; the pulsations were scarcely perceived, even by the ear, and the temperature of the limb, previously low, began to rise. On the 6th March, the circumference was again diminished by an inch, and the integuments were losing their bluish colour. After eight months' treatment no pulsation was felt in the tumour, and the limb was moved with facility. Compression was now applied by means of a bandage, and frictions of hydriodate of potash were employed to the engorged cellular tissue. At the end of eleven months the limb was reduced to nearly its natural size, but no pulsations were yet felt in the ham or leg. The patient was allowed to rise and walk a little on crutches, but the swelling and pain being somewhat increased, he was again restricted to his couch. After this, the improvement continued, but more slowly. The application of ice was continued unceasingly *for two years*, at the end of which time the tumour was hard and flattened, and the thigh had returned to its natural state. He was now allowed to walk. Pulsations were now felt in the lower third of the femoral artery and in the anterior tibial. In short, the aneurism was cured, and the patient was shortly afterwards discharged from the hospital, scarcely requiring the support of a stick in walking. During this long course of treatment the diet was light, the drink acidulated barley-water, and no medicine was administered.

*Gazette Médicale. September*, 1837.

---

### *On Dislocations of the Shoulder-joint.* By M. VELPEAU.

MUCH as has of late years been written on the subject of luxations of the humerus, M. Velpeau considers that, both as regards their diagnosis and mode of reduction, much remains to be done. The paper which we are about to analyze contains principally the result of his own experience on the subject, in which he has endeavoured, though not to settle all doubtful points, to contribute something to their explanation.

The question as to whether the humerus can or cannot be dislocated upwards or downwards, he regards as one merely of words; the careless use of terms having been the origin of misapprehension. There are only two principal directions in which the humerus can be dislocated,—the *antero-internal* and *postero-external*, as it respects the glenoid cavity; i. e. on the side of the axilla and on that of the sub-spinous fossa.

I. The *postero-external, sub-acromial, sub-spino*, is now admitted beyond a doubt.

II. With regard to the subject of *incomplete* luxations, M. Velpeau considers that there is the same confusion as that above referred to. If, by an incomplete luxation, is meant that the cartilaginous surface of the head of the humerus has but half escaped from the glenoid cavity, its existence must doubtless be denied; but if by incomplete it is to be understood that the head of the humerus is detained by some portion of its anatomical neck upon the border of the glenoid cavity, the possibility, the existence, and even the facility of such a dislocation should not be denied. This should not be regarded as a complete luxation; because in it there is manifestly escape of the cartilaginous hemisphere and of a portion of the bony circumference which separates it from the surgical neck of the bone, whilst, in the incomplete form, the osseo-fibrous structure remains partially within the capsule. Of incomplete luxation, so understood, M. Velpeau believes that he has met with three cases; two outwards and one inwards. Very many surgeons have admitted incomplete luxations towards the axilla as of possible occurrence. M. Velpeau has related a case, with its dissection, illustrative of this form of accident. Concerning these cases, however, although in this instance it is contrary to the author's opinion, there is the doubt whether the situation in which the bone was found after death was not acquired subsequent to a complete dislocation of the head of the bone.

III. The *antero-internal* or *axillary* luxations are thus divided by M. Velpeau: (1) sub-pectoral; (2) sub-scapular; (3) sub-clavicular. These forms, he adds, have a mechanism, a seat, and a mode of reduction, which it is often of advantage not to confound.

(1) The *sub-pectoral* is that form in which the head of the humerus is situated in the hollow of the axilla, between the sub-scapular and pectoral muscles. This is the luxation downwards of surgeons. (2) In the *sub-scapular*, the head of the bone, lodged in the axillary fossa of the scapula, is separated from the hollow of the axilla by the sub-scapular muscle. (3) In the *sub-clavicular*, the head of the humerus, situated near the root of the coracoïd process, is, as it were, bounded beneath by the superior part of the sub-scapular muscle. The sub-pectoral dislocation will chiefly happen when, at the moment of displacement, the arm is elevated above the scapula beyond a right angle. The capsule is then torn beneath and inwards, so that the head of the humerus glides over the lower border, or through the least elevated fibres of the sub-scapular muscle, to place itself beneath the larger pectoral, in the hollow of the axilla. If the elbow is somewhat less elevated, or almost in the same plane with the shoulder, when the displacement, either by a direct or by an indirect cause, takes place, the sub-scapular luxation must be expected. As to the sub-clavicular luxation, it is scarcely possible but as a consequence of force, exercised upon the elbow or hand from below upwards and from without inwards, or, indeed, after violence or blows effected upon the upper third of the humerus in the same direction, the elbow being confined by some other force. These three kinds of dislocation present certain differential signs, which, first of all established in the living subject, and afterwards by direct experiment upon the corpse, have appeared to vary but in a very trifling degree; thus—

| (1) *Sub-pectoral.* | (2) *Sub-scapular.* | (3) *Sub-clavicular.* |
|---|---|---|
| Pectoralis major more or less raised; sub-clavicular hollow preserved. | Pectoralis major scarcely raised; sub-clavicular hollow almost effaced. | Pectoralis major thickened without being elevated; relief in the situation of the sub-clavicular hollow. |
| Acromion very prominent; depression of deltoid considerable. | Acromion less prominent, and deltoid less stretched. | Acromial projection and depression of the deltoid more evident behind than before. |
| Head of humerus almost naked beneath the skin in axilla, which it fills; easily felt through pectoralis major from before backwards. | Head of humerus separated from skin in axilla by a thick layer of tissues; can be but with difficulty traced through the pectoralis major: the fingers can be passed between it and the inner parietes of the thorax. | Head of humerus at the top of the axilla, and difficult to feel; appreciable in front through the elevated portions of the deltoid and pectoral muscles. |
| Voluntary and communicated movements tolerably easy. Elbow separated from thorax, and carried somewhat backwards. | Movements of limb limited and somewhat difficult, producing a species of crepitation. Elbow separated from thorax, and carried somewhat forwards. | Movements painful, and almost impossible to effect; occasionally crepitation. |
| Arm from two to twelve lines longer than that of the opposite side; inferior angle of scapula raised considerably backwards, separated from the vertebral column. | Arm a few lines longer or shorter, or of the same length as that of the opposite side. Whole border of scapula elevated backwards. | Arm slightly bending backwards, but scarcely separated outwards from the trunk; shorter than that of the opposite side; posterior border of scapula more projecting superiorly than below. |

The above must rather be regarded as general rules than as an absolute diagnosis. The three species of luxations above described may differ by such trifling circumstances as to render an absolute definition impossible. The elevation of the inferior and superior angle, of the whole posterior border of the scapula, and its distance more or less from the vertebræ, appear to deserve a degree of attention which they have not yet received. The species of crepitation alluded to in the second and third forms of luxation is not found in the sub-pectoral. Occasionally the sound is very loud. In order fully to appreciate it, the shoulder should be grasped by the hand resting upon the acromion, whilst the limb is quickly rotated by the other hand. This symptom should be very carefully studied, as it may give rise to the suspicion of fracture which does not exist; but, instead of the fine and sonorous crepitation of bone, it is rather a deep muffled crackling of the cartilages.

The change in the *length* of the arm is a subject of much importance, considering what has been lately advanced on the subject. M. Malgaigne has maintained that it is always longer when dislocated, and that it cannot be shorter; and M. Sedillot, that the want of such elongation would be inexplicable. Velpeau was at first disposed to coincide in these opinions. Its value, if true, as a means of distinguishing dislocation from fractures of the neck of the humerus would be very great, because in the latter the arm is almost always shortened: but he did not find that it was in accordance with fact, and others, to whom he communicated his experience, were equally disappointed. A pupil, who performed twenty experiments upon the dead body, with the intention of ascertaining the truth, came, indeed, to a contrary conclusion; i. e. that, in luxations of the humerus, the arm is almost always shortened. M. Velpeau believes that he has not only ascertained that the arm is not always lengthened, but the conditions to which either its lengthening or shortening is ascribable. Having luxated the humerus in the dead body, he has assured himself that (1) there is elongation of some lines, from half an inch to an inch, but never to two inches, as has been inadvertently said, in the sub-pectoral luxations; (2) the length is almost the same, from two to three lines, more or less, than that of the healthy side, in the subscapular luxations; (3) there is a shortening, varying from a few lines to an inch, in the sub-clavicular dislocations. Applying these results to clinical practice, he has found them hitherto universally confirmed.

M. Sedillot, renouncing the idea that elongation was necessary, stated, after having seen some of Velpeau's cases, that the dislocated arm ought always to be shortened when it is held in a horizontal position; i. e. at a right angle with the body, whilst the measurement is being made. The head of the humerus, having slidden towards the subscapular fossa, should in this case be placed upon a plane posterior to that of the glenoid cavity, and thus render the shortening of the limb necessary. But a condition apparently so true in theory is contradicted by experience. A case is related of a sub-scapular luxation: when measured whilst parallel to the trunk, the luxated arm was of the same length as its fellow; elevated to a right angle with the trunk, it was found to be about four lines longer. The measurement was repeated many times, and in all possible manners, but always with the same result. Hoping that this case might be justly regarded as exceptional, the enquiry was repeated in other cases, but with no different effect. In six individuals who allowed their arms to be measured in this position, the limb was found to be lengthened; or, at least, not to present any shortening. In one of these, the lengthening was nearly an inch. This fact was attested before very many witnesses. It is difficult to explain. Unless it be admitted that, in elevating the arm, the acromion and the glenoid cavity are depressed backwards, so as to increase by one or two inches the space which naturally separates the anterior extremity of the spine of the scapula from the epicondyle, the author does not see how to explain the elongation.

The application of the foregoing observations to practice is especially important, as it concerns a choice of the various methods of reduction which have been so greatly commended. It is from not having paid sufficient attention to the distinctions above established, that practitioners are so little agreed as to the relative or absolute value of each mode of reduction. By excluding all methods but one, it is impossible to obtain any very exact inferences; for the method of reduction best suited to one kind of luxation may be, of all, the least proper for any other. Regarding the subject thus, M. Velpeau concludes that (1) the vertical extension, the limb being raised to the side of the head, is best accommodated to the sub-pectoral luxations; (2) the horizontal extension succeeds best in the sub-scapular luxations; (3) in the sub-clavicular, oblique extension downwards, and then horizontally, is the best suited to the object in view. In the majority of simple cases, each one of these methods may suffice; and such is the reason that they have all been adopted.

The elevation of the arm, which, in the sub-pectoral luxation, brings, without effort, the head of the humerus beneath the border of the sub-scapular muscle, towards the anterior border of the scapula, and which then allows it to enter into the glenoid cavity, does not present the same advantages as it regards the other luxations. This movement, which the head of the humerus suffers, favouring, for example, the folding or rolling up of some of the fibres of the sub-scapular muscle and capsule between the border of the glenoid cavity and the anatomical neck of the humerus would, in the sub-scapular and sub-clavicular luxations, be but an impediment to the end at which the surgeon aims; but the extension at first somewhat oblique, and afterwards horizontal, enable the end of the bone to find with facility the aperture in the capsule, if the luxations be of the other forms. Conformably with these principles, M. Velpeau employs a certain mode of reduction in a certain species of dislocation; but, even in sub-pectoral luxations, the horizontal extension succeeds almost as well as the vertical; and, although the latter method may be preserved in practice, it will be probably but as exceptional.

In the latter part of his paper, M. Velpeau makes some observations on direct luxations of the humerus. A case is given, in which it was certain that, when the dislocation occurred, the arm was applied to the side of the thorax, and that it took place in consequence of direct pressure upon the shoulder. Great contusion and enormous ecchymosis beneath the acromion, together with the absence of all injury of the elbow or hand, would have sufficed to prove the mode in which the dislocation occurred, even if the account of the individual himself had not been conclusive. This is a rare case, and valuable as evidence against those who deny the possibility of dislocation occurring in such a manner; particularly as, from the swelling and species of crepitation accompanying it, together with the facility with which blows upon the shoulder fracture the neck of the humerus, the opinion would generally have preponderated in favour of such a fracture, rather than of a dislocation the possibility of which is scarcely credited.

*Archives générales de Médécine.* Tome ii. *Juillet,* 1837.

---

### *On sudden Dilatation, a New Method employed by* PROFESSOR LALLEMAND *in destroying Strictures of the Urethra.*

THE method employed by M. Lallemand is opposed to the general practice in similar cases; but if his account of its success be correct, it strongly recommends itself to the notice of surgeons.

After having well determined the existence of one or more strictures in the urethra; after having above all things ascertained that no acute inflammatory condition exists, the patient is prepared for the treatment by some general or local baths, by a mild regimen and cooling drinks. The first gum elastic sound is then introduced. It is rare that with perseverance and care, No. 3 cannot be passed; but rather than over-fatigue the patient it is preferable to pass a sound of smaller size or a piece of catgut. When the sound has passed the obstruction, it must be fixed in the usual manner, rest must be enjoined and abstinence. The cooling drinks should be continued. If the sound has been introduced with facility, the patient may be again visited in two hours' time, and by gently drawing the instrument backwards and forwards it may be ascertained whether it is free or the contrary in the urethra. In the former case, it must be withdrawn, and a sound of the next size introduced: in the latter, it must be allowed to remain for at least an hour. The sounds must be afterwards changed every two hours, or even every hour; taking care however that no sound is removed, until it ceases to be retained tightly by the canal of the urethra. If it is found that the instruments pass easily, instead of passing regularly through the series of sounds, one may be here and there omitted; thus, Nos. 5 and 7, or 7 and 9 may be employed, omitting Nos. 6 and 8. In the course of two or three days, and sometimes in the course of twenty-four hours, the surgeon is enabled, by these means, to introduce No. 14, and to dissipate the engorgements which frequently diminish the urethra in the proportion of three-fourths of its caliber. As soon as the last sound is withdrawn, the canal returns to its ordinary diameter; but it is an essential part of the treatment that it should be submitted to the forcible distension produced by No. 14, otherwise the stricture would not be destroyed. The sounds when removed are discovered to be covered with mucosities. This is an exudation both necessary and useful. But if the presence of the sounds should cause too great a degree of irritation, this may require local depletion, &c.

When, after some instruments have been introduced, considerable difficulty is found in reaching the bladder, and particularly when the disease is of ancient date, it is possible that in addition to the submucous engorgement, there exist actual callosities. In this case, the patient should be allowed to rest for a day or two, and cauterization should be then had recourse to. This may be done in the ordinary manner, and it is rare that one cauterization will not be sufficient to perfect the cure, already very advanced by compression. A few days after the cauterization, some small eschars are evacuated from the urethra together with the urine. The only precaution required then is, that, during two or three weeks, an elastic bougie of the size No. 8 or 9 should be introduced every night into the bladder. This precaution, to which indeed the patient should submit whether there has been necessity to employ caustic or no, suffices to preserve afterwards the ordinary diameter of the canal.

Dilatation thus employed possesses the advantage over the other means of being equally efficacious, and less subject to accidents; and, besides, its greatest merit is to shorten the treatment very materially, and to facilitate, when it is necessary, the employment of cauterization. Patients also submit to it with greater willingness; for it is well known that the abuse of cauterization has lately given rise to considerable prejudice. It is now more than three years that Professor Lallemand has employed with success this new method, distinguished by the name of *sudden dilatation,* and many to whom it has been communicated have found reason to congratulate themselves on being acquainted with it.

*Gazette Médicale.* No. 26. *Juin,* 1836.

---

### *On a new Method of Treating the Hectic Fever which follows the Operation for Empyema.* By M. Fuster.

M. Recamier, considering the hectic fever which follows the operation of empyema to be caused by an alteration of the pus consequent on its mixture with the external air, has proposed to inject into the pleural cavity, immediately after the operation, a certain quantity of water, at the temperature of 28 or 30° R. (about 98° F.) to fill the void which would otherwise be occupied by atmospheric air. He has not found pain or inflammatory reaction to result from the presence of the water, which must be injected in less quantity in proportion as the compressed lung regains its normal dimension. The use of injections is contraindicated when the pleura is in an irritable state, or when tubercles exist in the lungs. "In fact," says M. Fuster, "this practice is only applicable when the pleura and lungs have returned to a healthy state, and we have only to treat the fluid effusion, the result of an old affection." If these conditions are necessary to the success of the practice, it will be, indeed, rarely applicable; for we never find the pleura in a healthy state when purulent effusion exists, and the patient is much more frequently destroyed by the disease of the serous membrane than by the existence of pus in the cavity. It is said that M. Recamier has treated many cases successfully by this method; but, as no numerical details are given as to the proportions of successes and failures, we know not what degree of confidence may be placed in the statement. We have, since the year 1832, made trial of the method of M. Recamier, not in cases of empyema, but for the hectic fever which is consecutive on large suppurations of the extremities, and among others for deep abscess of the thigh; but, notwithstanding the precautions used, we have not been able to prevent the bad effects of absorption, which have proved fatal more or less quickly.

*La Presse Médicale.* 1837.

---

*Fracture of the Ribs by Contre-coup.* By Dr. MONDIERE.

THE author relates this case as unique. A robust man, aged forty-five, fell from a height of about forty feet, upon some ground recently dug. When he fell, his hands were crossed over the chest, the legs flexed on the thighs, and the thighs on the pelvis; the head also was inclined upon the thorax; so that he fell flat upon the right side, the only part which came in contact with the ground. He lost almost wholly the power of motion of his lower limbs; the sensibility of which was, nevertheless, very much increased. The fourth, fifth, and sixth ribs of the left side were fractured transversely, at a very slight distance from their cartilages. The integuments covering the broken ribs were not in the slightest degree bruised; and the manner in which he had fallen having been ascertained from others besides himself, no other inference was admissible than that the fracture was a result of *contre-coup*. On that part of the right side on which he had fallen was an extensive ecchymosis, exactly opposite the seat of the fractures. The patient perfectly recovered. *Archives générales de Médecine.* Tome ii. *Juillet,* 1837.

---

*On the Treatment of Wounds by the Affusion of Cold Water.* By M. GODIN.

IN the British and Foreign Medical Review, for January, 1837, we noticed the observations of M. Josse, of Amiens, on the treatment of many diseases by the affusion of cold water. The author of this paper, a pupil at the Hôpital Neckar, relates several cases of lacerated wounds treated in this manner successfully by M. Berard. The views of M. Berard as to the therapeutical effects of the cold affusion are thus explained:—After relating a case of very extensive lacerated wound, which cicatrized rapidly, M. Godin continues,

"I think that this favorable result is in part to be attributed to the continuance of a very elevated temperature during the month of July and part of August. It may perhaps appear strange that I should attribute an advantageous effect to *heat* after having established that of *cold* applied by means of irrigation, but we must here make an important distinction. *Cold* can be of no advantage in promoting the cicatrization of wounds, and it is not with this view that M. Berard employs it locally in the system of continuous irrigation. Every contused wound must suppurate; suppuration cannot be established without a reaction of more or less intensity; and it is in order to restrain this reaction within moderate bounds, to prevent the extension of inflammation to neighbouring parts, that the affusion is to be employed. It becomes useless when suppuration is well established."

*Archives gén. de Méd. Mars,* 1837.

---

*Method of Promoting a Flow of Blood after Venesection.* By Dr. BURDACH, of Triebel.

IT is not unfrequently the case, when venesection has been performed, that the vein is found empty, and that no blood can be drawn. Under these circumstances compression and friction are often of no avail, and the only sure means of exciting the venous circulation is to press the vessels of both arms, and by that means to cause a sympathetic excitement of the corresponding venous trunks of both sides: thus a flow of blood is rapidly effected, and it may be drawn to any extent. When a vein has been opened without effect, the other arm should also be bandaged above the elbow, and a little tighter than if it was intended to perform venesection in it: the consequence will be, that its veins will swell from above downwards to the fingers' ends. Very shortly the veins of the other arm will fill in the same manner. As soon as the state of congestion is indicated by the feeling of deadness in the fingers' ends, the bandage should be loosened to such an extent that it may be nearly removed; then the blood should be gently directed by the pressure of the thumb to the orifice made by the lancet, from which, in this way, a plentiful and continuous stream may be made to flow, to be regulated by the compression or relaxation of the bandage. It is essential to the success of this experiment that the compression, which is not merely mechanical, and which acts in a peculiar manner upon the venous trunks, should be properly regulated, increased or diminished. This practice will also be found very useful in bleeding those women in whom, from their corpulency, the superficial veins of the arm are found with much difficulty, and furnish but little blood. Only in these cases more time is required; seeing that the very small veins have not only to fill but to expand; whilst, in those above mentioned, the caliber of the vessels is sufficiently large, and they are only empty from derangement of the small circulation, or from a primary, morbid inactivity of the peripheral venous system. This method of promoting the abstraction of blood may be often practised, with a good result, on the feet; but here its success is not so certain, probably because the circulation is not so active, and therefore to obstruct it not so efficacious as in the arm.

*Graefe und v. Walther's Journal.* B. 22.

---

### *On the Use of Soot in Diseases of the Bladder.* By M. André Gibrin.

This gentleman has transmitted to the Academy of Medicine six cases of chronic inflammation of the bladder, in which *soot* was beneficially employed. In two of these, which proved fatal, and in which ulceration of the fundus existed, the symptoms were alleviated: the others were cured. The symptoms were occasional retention of urine, pain at the hypogastrium, frequent desire to make water, a turbid and fetid state of the secretion, which was mixed with mucus and sometimes with blood, and deposited a viscous sediment which adhered to the sides of the vessel. Having tried various modes of treatment without success, M. Giboin determined to have recourse to the decoction of soot, from the external application of which he had derived great advantages; but he does not state how, or in what diseases, he had employed it.

He took from the chimney two ounces of compact soot, broke it up, washed it, and boiled it in a pound of water. The decoction was filtered through paper, and injected into the bladder twice a day. The good effect followed so closely the administration of the remedy, that it was impossible to be mistaken as to the cause. The pain ceased, and the patient obtained sleep, to which he had been for some time a stranger. The urine gradually became clear, and recovered its natural appearance. The two patients in whom ulceration of the bladder existed obtained much relief from the injection, and, on examination, the ulcerations were found to have lost that livid appearance which they usually present.

*Bulletin de l'Académie.* 15 *Mars,* 1837.

---

### *On the Use of Chloride of Lime in Wounds attended with much Pain.* By Dr. Chopin.

In wounds produced by contusion, laceration, or by the explosion of gunpowder, where there is much pain, speedy and certain relief, says Dr. C., is produced by chloride of lime. That this relief is not the effect of cold or any other cause than the chloride in solution, the author is convinced by many experiments. Charpie, moistened with the same solution, has been also found a useful application in relieving the pains which sometimes follow delivery, which depend on small excrescences in the vagina. That such is frequently the case, Dr. C. is convinced from repeated examination. Excoriated breasts are most efficiently treated by the use of the same external application.

*Jahrbücher der in-und-ausländischen gesammten Medicin.* Heft i. 1837.

### *Treatment of incised Wounds by Means of the Membranes of the Egg.* By Dr. Heussner.

Dr. H. commends a popular remedy, not generally known, as a means of keeping the edges of wounds in contact. The parents of a child, who had cut its lip considerably by a fall, were unwilling that sutures should be employed, and the constant escape of saliva from the wound prevented the adhesion of the ordinary plaster. The mother of the child broke an egg, and took from it the thin membrane which lines the shell. This was placed obliquely across the wound in several strips. They adhered well, quickly dried, and united so closely to one another, that the borders of the wound came into close contact, preventing the escape of saliva, and favouring the healing process, which was complete in a few days.

*Wochenschrift für die gesammte Heilkunde.* No. 25. 1837.

